# Modeling HSV-1 Latency in Human Embryonic Stem Cell-Derived Neurons

**DOI:** 10.3390/pathogens6020024

**Published:** 2017-06-08

**Authors:** Aldo Pourchet, Aram S. Modrek, Dimitris G. Placantonakis, Ian Mohr, Angus C. Wilson

**Affiliations:** 1 Department of Microbiology, New York University School of Medicine, New York, NY 10016, USA; pourchet@upenn.edu (A.P.); Ian.Mohr@med.nyu.edu (I.M.); 2 Department of Neurosurgery, New York University School of Medicine, New York, NY 10016, USA; Aram.Modrek@med.nyu.edu (A.S.M.); Dimitris.Placantonakis@nyumc.org (D.G.P.); 3 Kimmel Center for Stem Cell Biology, New York University School of Medicine, New York, NY 10016, USA; 4 Brain Tumor Center, New York University School of Medicine, New York, NY 10016, USA; 5 Perlmutter Cancer Center, New York University School of Medicine, New York, NY 10016, USA; 6 Neuroscience Institute, New York University School of Medicine, New York, NY 10016, USA

**Keywords:** herpes simplex virus, HSV-1, alphaherpesvirus, human, primary neurons, latency, reactivation, embryonic stem cells

## Abstract

Herpes simplex virus 1 (HSV-1) uses latency in peripheral ganglia to persist in its human host, however, recurrent reactivation from this reservoir can cause debilitating and potentially life-threatening disease. Most studies of latency use live-animal infection models, but these are complex, multilayered systems and can be difficult to manipulate. Infection of cultured primary neurons provides a powerful alternative, yielding important insights into host signaling pathways controlling latency. However, small animal models do not recapitulate all aspects of HSV-1 infection in humans and are limited in terms of the available molecular tools. To address this, we have developed a latency model based on human neurons differentiated in culture from an NIH-approved embryonic stem cell line. The resulting neurons are highly permissive for replication of wild-type HSV-1, but establish a non-productive infection state resembling latency when infected at low viral doses in the presence of the antivirals acyclovir and interferon-α. In this state, viral replication and expression of a late viral gene marker are not detected but there is an accumulation of the viral latency-associated transcript (LAT) RNA. After a six-day establishment period, antivirals can be removed and the infected cultures maintained for several weeks. Subsequent treatment with sodium butyrate induces reactivation and production of new infectious virus. Human neurons derived from stem cells provide the appropriate species context to study this exclusively human virus with the potential for more extensive manipulation of the progenitors and access to a wide range of preexisting molecular tools.

## 1. Introduction

Herpes simplex virus type 1 (HSV-1, Human alphaherpesvirus 1) infects >70% of the human population, establishing a lifelong infection of the peripheral nervous system (PNS), termed latency [1]. Recurrent reactivation of latent virus ensures transmission and in some can also cause diseases such as painful oral lesions, post-herpetic neuralgia, burning mouth syndrome, Bell’s palsy, and blindness [2,3]. Spread of the reactivated virus into the central nervous system (CNS) may lead to more severe disease outcomes, such as birth defects, myelitis, and lethal viral encephalitis.

To date, rats, mice, guinea pigs, and rabbits have served as the principle models to study HSV-1 latency and pathogenesis, both as live-animal infections (in vivo) and as the source of cultured neurons or explanted ganglia (in vitro) [4,5,6]. A wealth of valuable information has come from these studies, but it is clear that aspects of infections in humans, the only natural host of HSV-1, are not necessarily recapitulated [6]. For example, in humans, HSV-1 reactivation episodes are very frequent and are further accentuated by environmental insults, such as exposure to ultraviolet light or psychological and physiological stresses. Spontaneous and systemic stress-induced shedding does occur with a limited number of HSV-1 strains in the rabbit ocular model [7,8] but there are logistical issues that have limited the use of rabbits for most studies. Mice do not reproduce the pathophysiology of human disease and show significantly lower levels of spontaneous reactivation for most viral strains [9,10,11,12,13,14,15,16,17]. It seems that latency is more tightly controlled in rodents than in humans, possibly a combination of a reduced ability of rodent cells to support productive (lytic) viral replication and increased effectiveness of their innate and acquired immune systems to suppress reactivation. There are several examples of specific virus-host interactions that are effective in human, but not rodent cells. HSV-1 ICP47 protein is a poor inhibitor of the transporter associated with antigen processing (TAP) in rodent cells [18]. Likewise, the association of the HSV-1 lytic cycle initiator protein VP16 with its host DNA-binding partner Oct-1 is also significantly reduced in rodent cells [19,20].

These concerns aside, we and others have used infection of primary neurons isolated from the superior cervical ganglia (SCG) of prenatal rats and post-natal mice to delineate the contribution of host signaling pathways to the control of HSV-1 latency [21,22], to characterize the viral transcriptional response during latency and reactivation [19,23,24] and to define the neuron autonomous action of interferons in suppressing reactivation [25]. The methodology to establish latency with the wild-type virus in SCG neurons has been described in detail [21,26,27], and builds on the pioneering work of Christine Wilcox and Eugene Johnson in the late 1980s [28,29,30]. Sympathetic neurons from the SCG are used because the cultures are relatively uniform and contain fewer non-neuronal cells than cultures obtained from other ganglia, however, the methodology has been successfully adapted to the dorsal root (DRG), geniculate, and vestibular ganglia [28,31,32]. Despite these recent advances (reviewed in [5,33,34]), the limitations of using rodent, rather than human, neurons remain and can potentially mask or over-emphasize the contribution of individual virus-host interactions. As such, there is a need to explore these processes in a human neuronal context.

The use of sensory and cranial nerve ganglia isolated from fresh human cadavers has been investigated [35], but it is very difficult to achieve material of sufficient consistency and the ganglia from adult donors are often infected already with HSV-1 and/or varicella zoster virus (VZV) [36,37]. A more practical alternative would be to infect human neurons generated in vitro either from embryonic stem (ES) or induced pluripotent stem (iPS) cells. This approaches have been explored to good effect in recent studies of VZV latency, which is reproduced poorly in non-human systems [38,39,40,41,42].

Embryonic stem cells are derived from the blastocyst, support unlimited self-renewal, and can be differentiated into cells and tissues of all three germ layers [43]. Furthermore, a number of human ES cell (hESC) lines have been identified that can be differentiated into functional neurons [44], providing valuable tools for neurological disease research and as an essentially limitless source of transplantable cells for regenerative medicine. We took advantage of this powerful technology to produce human embryonic stem cell (hESC)-derived neurons that can be infected with HSV-1 under conditions that support a stable non-productive infection closely resembling latency. We found that the combination of low viral inoculum and treatment with both acyclovir and interferon-α at the time of infection effectively suppressed productive (lytic) cycle gene expression and protected the neurons from the cytopathic effects associated with viral replication. Cultures carrying viral genomes could be maintained for several weeks in the absence of the antiviral agents and subsequently induced to reactivate and produce new infectious virus by treatment with a generalized histone deacetylase inhibitor. This provides an attractive model to study both viral and host determinants of latency and reactivation, especially interactions that are species-dependent. One of the drawbacks of using neurons isolated from rats is the scarcity of well-characterized molecular tools, such as antibodies. This is not the case for human neurons. Lastly, because the neurons are generated in vitro it may also be possible to apply gene-editing strategies to directly modify the neuronal environment prior to infection.

## 2. Results

### 2.1. Stepwise Differentiation of the Hes5::GFP Human Embryonic Stem Cell Line into Neurons

**Figure 1:**
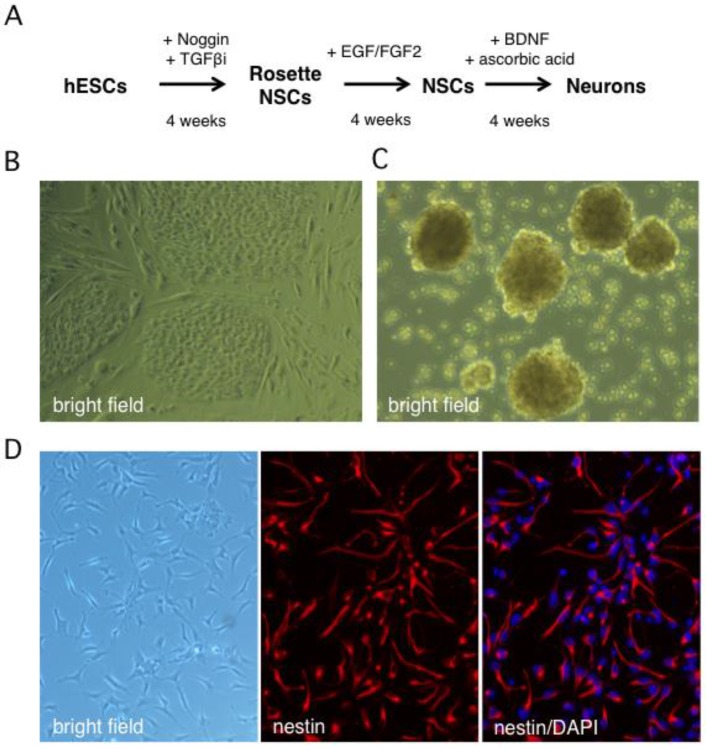
In vitro derivation of human neural stem cells by differentiation of the Hes5::GFP human embryonic stem cell line. (**A**) Schematic showing the multistep neural induction protocol. TGFβi stands for TGF-β receptor I inhibitor (**B**) Bright field image of human embryonic stem cell (hESC) colonies cultured on mouse embryonic fibroblasts prior to reaching confluence. (**C**) Bright field image of rosette NSCs derived from dissociated hESC colonies cultured in neural induction media. (**D**) Phase contrast and indirect immunofluorescence images of NSC cultures grown on poly-l-ornithine/laminin-coated dishes in neural stem cell media and probed with an antibody against nestin, a neural stem cell marker. Nuclei were visualized with DAPI.

**Figure 2:**
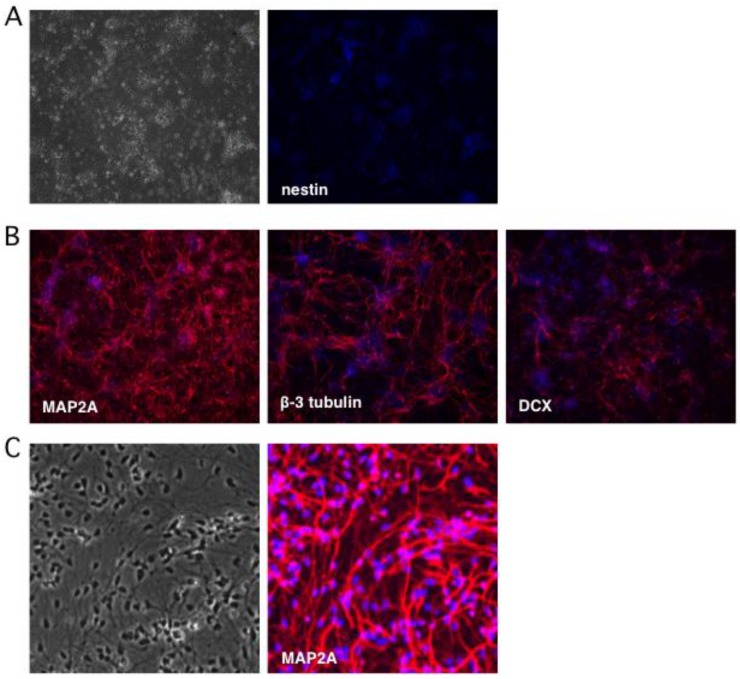
Neuronal cultures lose expression of the progenitor marker nestin and gain expression of multiple neuronal markers. Neuronal differentiation was monitored by indirect immunofluorescence microscopy using antibodies to (**A**) nestin, a marker of NSCs, and (**B**) neuronal markers MAP2A, β-3 tubulin (Tuj1), and doublecortin (DCX). (**C**) Higher magnification imaging reveals a relatively homogeneous population of neurons expressing MAP2A. Nuclei were detected with DAPI.

Human neurons were generated from the Hes5::GFP hESC line [45] using the stepwise differentiation protocol shown schematically in Figure 1A. In this scheme, hESC colonies are induced to differentiate into neural stem cell (NSC) rosettes, which are multipotent early neural progenitors reminiscent of the neural plate. After dissociation, rosette NSCs are plated as adherent NSCs (Figure 1B,C), which resemble neural progenitors in the developed central nervous system and can be further differentiated into neurons [45]. The Hes5::GFP reporter specifically identifies GFP+ rosette neural stem cells (NSCs), but GFP is turned off in adherent NSCs and neurons [46]. We monitored the efficiency of differentiation from hESCs to NSCs by indirect immunofluorescence (IIF) microscopy with an antibody to the type VI intermediate filament protein nestin, a neural stem cell marker [47,48]. After four weeks, close to 100% of surviving cells had become nestin positive, indicating efficient differentiation (Figure 1D or Figure 2A). An important advantage of this protocol is that it omits the need to generate neurospheres, free-floating clusters of neural stem cells, which can be a source of heterogeneity due to uneven exposure of progenitor cells to morphogens in the media and to differences in cell-cell contacts [49]. This is critical because undifferentiated progenitor cells may differ from neurons in their susceptibility to infection by HSV-1.

To monitor the terminal differentiation step, indirect immunofluorescence (IFF) was performed using antibodies as markers of mature neurons: microtubule-associated protein 2A (MAP2A) was detected in the cytoplasm of 95–100% of the differentiated neurons, while β-3 tubulin (also known as Tuj1) was observed in 75% (Figure 2B). Thirty percent of neurons were positive for microtubule-associated protein doublecortin (DCX), a marker of neuroblasts and immature neurons [50,51]. Transmitted light imaging revealed homogeneous cultures of neuron-like cells with extensive neurites and nearly all of the cells were MAP2A positive (Figure 2C). Based on these observations we estimate that undifferentiated progenitor cells comprised only a very minor fraction of the cultures, which otherwise consist of terminally-differentiated neurons.

### 2.2. Human Neurons Are Permissive for HSV-1 Replication

**Figure 3:**
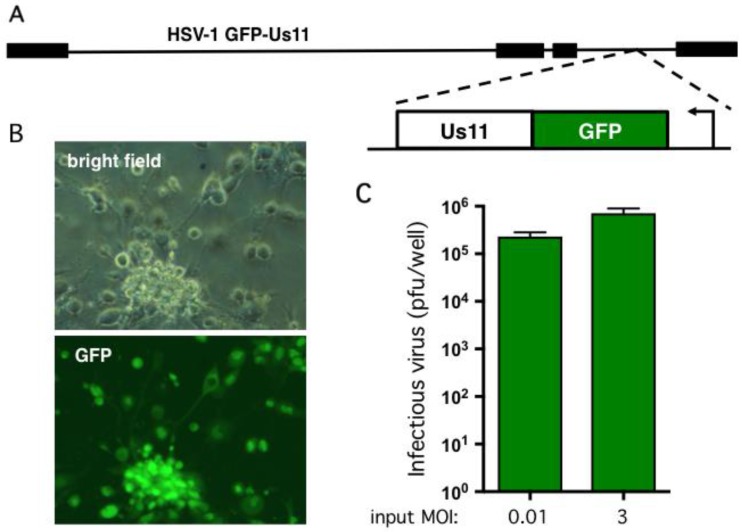
hESC-derived neurons support productive HSV-1 replication. (**A**) Schematic showing HSV-1 GFP-Us11 (Patton strain), a recombinant expressing enhanced GFP fused in frame to the N terminus of the true late protein Us11 [52]. (**B**) Bright field and fluorescent imaging of neurons infected at MOI = 0.001 and imaged by light microscopy after 4 days. The majority of the neurons exhibited obvious cytopathogenic effects and expressed the true-late GFP-Us11 fusion protein indicative of active viral replication. (**C**) Multi-cycle replication assay. 1 × 10^5^ neurons were infected at either MOI = 0.01 (1 × 10^3^ pfu) or MOI = 3 (3 × 10^5^ pfu). Culture media was collected after a further four days and the yield of infectious virus determined by plaque assay on Vero cells. Values represent the total number of plaque forming units (pfu) per well and graphed as the mean ± SEM.

To assess viral replication in the hESC-derived neuron cultures we used a wild type HSV-1 (strain Patton) modified to express GFP fused with the true-late (γ2) protein Us11 [52] (Figure 3A). Expression of the GFP-Us11 fusion protein requires viral DNA replication and, as demonstrated previously, provides a simple, real-time indication of productive replication (wells score positive for green fluorescence) and non-productive infection (wells are negative for green fluorescence) [21]. GFP fluorescence is also a good indicator of infectious virion production and release as we were unable to detect plaque-forming units (pfu) in the culture media from wells that were negative for GFP fluorescence (data not shown). As shown in Figure 3B, infection at multiplicity of infection (MOI) of 0.001 pfu per neuron in the absence of an antiviral compound resulted in clear GFP expression and obvious cytopathic effects, such as rounding of the cell body in the majority of neurons. Within 1–2 days, neurons mostly detached from the plate. To quantify viral replication, 10^5^ neurons were infected at MOI = 0.01 and the growth media collected after four days. The number of plaque forming units (pfu) was determined by plating serial dilutions of culture media on Vero cell monolayers to detect non-cell associated virus (Figure 3C). This yielded a 230-fold amplification of the input virus in the lower MOI infection but as expected only a modest 2.4-fold increase at the higher MOI. Similar infection studies were performed with wild-type Patton virus and as expected did not result in visible GFP fluorescence upon infection but yielded similar titers of newly-replicated virus (data not shown). This demonstrates that the hESC-derived neurons are permissive for infection by HSV-1 and support productive viral growth. It also verifies that the Hes5::GFP transgene is not activated by viral infection.

### 2.3. Non-Productive Infections Can Be Established Using a Combination of Low Inoculum, Acyclovir, and High Dose IFN-α

A defining feature of HSV-1 latency is the suppression of productive replication and this must be achieved in any useful model. To explore this, we performed a series of trials to determine the relative importance of infectious inoculum, along with antiviral compounds acyclovir and interferon-α (IFN-α), in limiting viral replication in human neurons. In rat SCG-derived neurons, infection at MOI = 1 in the presence of 100 μM acyclovir is sufficient to suppress productive replication in the majority of cultures [21]. Unlike rat neurons, productive HSV-1 replication proceeded in human neurons under these conditions (data not shown), a reflection perhaps of the homologous neuronal environment.

**Figure 4:**
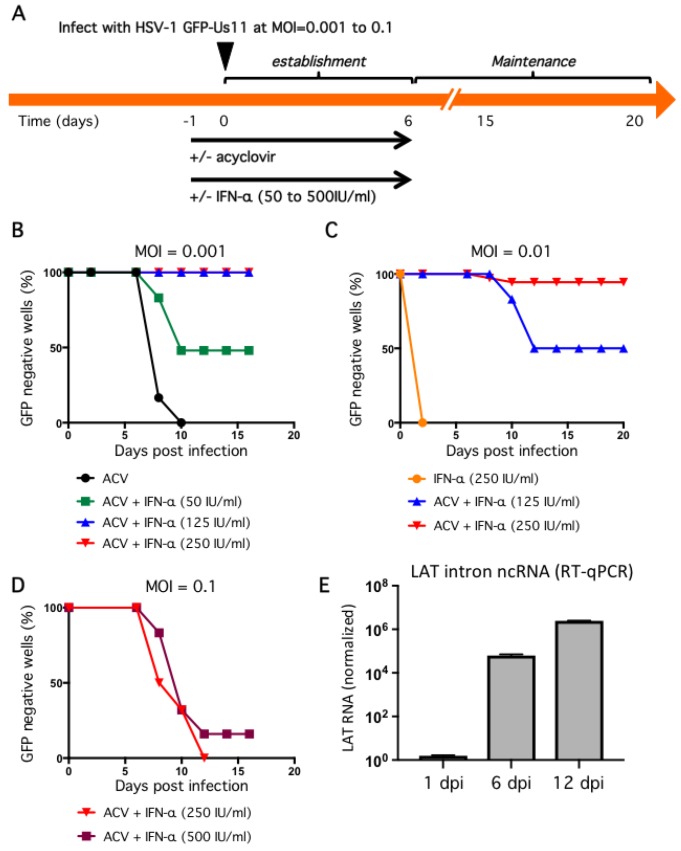
Dose-dependent suppression of productive replication by IFN-α in combination with ACV and low viral inoculum. (**A**) General infection scheme. Neurons were pretreated with 100 μM ACV and different doses of IFN-α the day before (designated day-1) infection with HSV-1 (day 0). (**B**–**D**) Evaluation of infectious dose and IFN-α treatment in the establishment of non-productive infections. Neurons were incubated with HSV-1 GFP-Us11 for 4 h at MOI = 0.001 (**B**), 0.01 (**C**), or 0.1 (**D**). After 4 h the media was replaced with fresh media and repeated every other day. ACV and IFN-α were included in the media as indicated until 6 dpi (‘establishment period’) at which point neurons were cultured in maintenance media lacking antiviral compounds and monitored daily for GFP fluorescence. Graphs show the percentage of GFP negative wells under each condition. (**E**) Neuron cultures were infected with HSV-1 GFP-Us11 at MOI = 0.01 in presence of ACV and 250 IU/mL IFN-α and RNA was collected at 1, 6, and 12 dpi and analyzed by quantitative reverse transcription PCR (qRT-PCR) using primers to the HSV-1 LAT intron. Values were normalized to host GAPDH mRNA.

We, therefore, opted to lower the initial inoculum and include a second antiviral agent. Infection at MOI = 0.001 in the presence of 100 μM ACV suppressed GFP fluorescence but this was not stable and all wells became GFP positive within four days of ACV removal (Figure 4B). Treatment with ACV together with 50 IU/mL IFN-α for the first 6 dpi was more effective, with 50% of wells remaining GFP negative by 16 dpi. This corresponds to 10 days without any antiviral agents. Increasing the dose of IFN-α to 125 IU/mL and higher still to 250 IU/mL was sufficient to suppress GFP expression in 100% of wells to 16 dpi, again corresponding to 10 days without any antiviral agents. Thus, the combination of low inoculum, and two antiviral agents during the establishment period, was enough to suppress productive cycle (lytic) gene expression as represented by the accumulation of the true-late protein GFP-Us11.

Although these conditions would be sufficient to study many aspects of HSV-1 infection, molecular studies of viral transcription and chromatin structure will likely benefit from maintaining a greater number of viral genomes and might be achieved through a higher initial infection dose. We therefore investigated infection at increased inoculum in the presence of ACV and IFN-α (Figure 4C,D). At MOI = 0.01, treatment with 250 IU/mL IFN-α without ACV was not sufficient to suppress viral productive replication and all wells were GFP-positive by 3 dpi. Again this was improved by the combination of ACV and 125 IU/mL IFN-α, with 50% of wells remaining GFP negative at 20 dpi. However, the combination of ACV and 250 IU/mL IFN-α resulted in 94% of wells remaining GFP negative for the course of the experiment. We also tested a ten-fold higher inoculum (MOI = 0.1) but found that after removal of the antiviral agents at 6 dpi, all wells quickly became GFP-positive, indicating a lack of sufficient host control (Figure 4D). Increasing the dose of IFN-α during the establishment period to 500 IU/mL had only a small effect.

One of the clearest molecular signatures of HSV-1 latency is the accumulation of one or more stable introns formed by incomplete splicing of the latency-associated transcripts (LAT) (reviewed in [53]). In latency models, the abundance of the LAT RNAs increases during the establishment of latency [11] and can be detected in neurons of ganglia from the sacral, thoracic, and lumbar regions of recently deceased humans [54]. To examine LAT expression in our model, neuron cultures were infected at MOI = 0.01 in the presence of ACV and 250 IU/mL IFN-α and total RNA was collected at different times and analyzed by quantitative reverse transcription PCR (qRT-PCR) using primers to detect HSV-1 LAT intronic RNAs (Figure 4E). Host glyceraldehyde 3-phosphate dehydrogenase (GAPDH) mRNA was assayed in parallel and used to normalize RNA recovery and processing. This revealed a substantial increase in LAT abundance between day 1 post infection and day 6, corresponding to the ACV and IFN-α enforced establishment period, increasing further by day 12, which corresponds to six days without any antiviral treatment. This confirms the presence of biologically-active viral genomes in GFP-negative cultures, as well as the capacity of these genomes to be transcribed after exposure to the antiviral agents.

### 2.4. Non-Productive Infections Reinitiate Productive Replication upon Treatment with Sodium Butyrate

**Figure 5:**
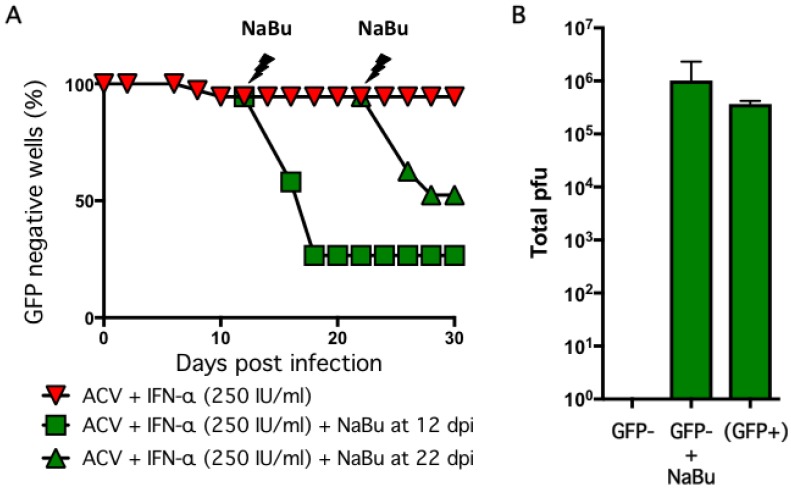
Reactivation of infected cultures using sodium butyrate. (**A**) Neurons infected with HSV-1 GFP-Us11 at MOI = 0.01 in presence of 100 μM ACV and 250 IU/mL IFN-α were treated with 5 mM sodium butyrate (NaBu) and monitored for GFP fluorescence. The NaBu was added at either 12 or 22 dpi. The percentage of GFP negative wells is shown. (**B**) Supernatants were collected at 12 dpi from GFP negative wells [GFP^−^] or NaBu treated and maintained for a further six days [GFP^+^ + NaBu] or spontaneously reactivated wells collected at 8 dpi [(GFP^+^)] and assayed in parallel for infectious virus using Vero cells. Values represent total number of plaque-forming units (pfu) per well and graphed as the mean ± SEM.

Arguably the most important feature of any latency model is the ability of the maintained episomes to reinitiate productive replication (reactivate) in response to a stress stimulus. To test this, we added sodium butyrate (NaBu) to wells that remained GFP negative after the establishment period. Butyrate is a general histone deacetylase inhibitor and this class of bioactive molecules have been used to reactivate latent herpesviruses, including HSV-1, in several in vitro and in vivo contexts [55,56,57,58,59,60]. Infected cultures were established by infection at MOI = 0.01 in the presence of ACV and 250 IU/mL IFN-α, the antiviral agents were removed at 6 dpi, and then maintained without further selection for up to 30 days (Figure 5A). At 12 dpi (i.e., six days after removal of the antiviral agents), NaBu was added to a series of wells and within four days, 74% of the wells had become GFP-positive, indicative of reactivation. NaBu was also added to a parallel set of GFP negative wells at 22 dpi (i.e., 16 days without selection) and of these 50% became GFP-positive within four days.

In addition to scoring green fluorescence, we measured infectious virus released into the culture supernatant by plaque assay on Vero cells (Figure 5B). At 12 days after infection, no plaque-forming units were detected in the culture media from GFP-negative wells demonstrating that the infectivity of the initial inoculum was lost. In contrast, a relatively high titer (10^6^ pfu/well) was obtained from wells treated with NaBu at 8 dpi and harvested at 12 dpi. This titer was comparable to untreated wells that were GFP-positive at 8 dpi (‘spontaneous reactivation’) and may reflect the finite capacity of the neurons in a well to produce virus.

Taken together these results indicate that infection of human neurons produced in vitro from a stem cell line with wild type HSV-1 can establish a stable non-productive infection that closely resembles latency as defined in humans and other models by the expression of abundant LAT RNA, the suppression of viral productive cycle gene expression and by retaining the ability to reactivate in response to a defined stimulus. Once infected, the cultures are stable and can be maintained without obvious cytopathic effects or evidence of active viral replication for a minimum of four weeks (Figure 5A).

## 3. Discussion

Humans are the natural host of HSV-1 but, for obvious reasons, there are both practical and ethical reasons that make it nearly impossible to study latency and reactivation in the human nervous system. Neurons isolated from rats or mice have provided effective surrogates but exhibit biological and technical limitations. Here we have explored the use of human neurons generated in vitro through the stepwise differentiation of a self-renewing hESC line. Since the neurons are generated in culture from proliferating progenitors, it should be relatively easy to scale up the cultures, opening up the exciting prospect of an accessible infection model that can be used for broader functional screens or biochemical analyses, the cost of which might be prohibitive in primary neurons.

We found the human neurons to be highly permissive for productive replication by wild-type HSV-1 and were, in fact, unable to control viral replication in the presence of the antiviral agent ACV, even at initial inoculums that were 100 to 1000-fold lower than used in the rat SCG neuronal model where ACV is sufficient [21]. This difference is likely a consequence of the more permissive human neuronal environment but, at this point, we cannot rule out other influences, such as neuronal subtype or the use of brain-derived neurotrophic factor (BDNF) rather than nerve growth factor (NGF) as the source of trophic support [61]. Similar to our studies, Sadaoka and colleagues found that ACV was not sufficient to control the productive infection of hESC-derived neurons by VZV, but were able to establish a persistent infection through the use of very low inoculums or by introducing the virus via the axons rather than the cell body [42].

There is a wealth of evidence showing that during natural infections, inflammatory cytokines, such as the type-I and type-II interferon (IFN), limit HSV-1 replication [62,63,64], acting directly on neurons themselves to suppress viral productive cycle gene transcription [25,65]. Thus, we reasoned that IFN might be able to complement or replace ACV in promoting the establishment of non-productive infections in our human hESC-derived neurons. In vivo, latency is established in the absence of antiviral agents, raising the question of why these are necessary in vitro. Neuron cultures differ from ganglia in many respects. Obviously, monolayers grown on plastic lack the three-dimensional arrangement of cell-to-cell contacts, and many non-neuronal cell types found in ganglia are absent in vitro. Indeed non-neuronal cells typically outnumber the neurons themselves and include satellite cells, fibroblasts, and various immune cells, all of which are potential sources of type-I and type-II interferons. These external influences may collaborate with the intrinsic control mechanisms of the neurons themselves to limit the expression of viral productive cycle genes and allow infected neurons to control incoming viral genomes by establishing the necessary repressive chromatin state [66]. IFN induces the expression of numerous antiviral factors (interferon-stimulated genes or ISGs) that include proteins that antagonize gene expression and promote the assembly of heterochromatin on viral genomes [67,68,69]. Compared to epithelial cells, neurons have a reduced capacity to produce IFN in response to HSV-1 infection [70], and during natural infections likely rely on other cell types to produce IFN and other inflammatory cytokines to which the neurons are responsive [65]. Pretreatment with a mixture of IFN-α and IFN-γ in combination with ACV is required to establish latency in neurons from the SCG of post-natal mice again perhaps reflecting differences in host species or neuronal maturity [23]. In other experiments we found that IFN-γ was not effective in controlling HSV-1 in our human neuronal cultures and was toxic to the neurons in combination with IFN-α [25].

When infected at a low inoculum (MOI = 0.01) in the presence of ACV and 250 IU/mL IFN-α our hESC-derived neurons were able to control wild type virus and establish a stable non-productive infection within a six-day period. After this time, both antiviral agents could be removed without the virus reengaging in productive replication unless the infected neurons were treated with a stress agent such as sodium butyrate. Stable infections could be maintained without any evidence of viral replication for more than 30 days. The ability to establish an infection in which the viral genome persists, but retain the capacity to reenter productive replication (i.e., reactivate), is a central tenet of accepted definitions of HSV-1 latency [66]. Infectious virus and GFP-Us11 expression, a marker of viral productive cycle (lytic) gene expression, were not detected after the establishment period, however the accumulation of LAT RNA, a molecular hallmark of latency, was readily observed [53]. Some of the next steps in characterizing this model will be to assess the structure of the viral episome during and after the establishment period and profile the expression of other latency-associated gene products such as the viral microRNAs [24].

An issue for many in vitro infection models is heterogeneity within the cultured cell population, which can influence the behavior of the virus [71]. The presence of multiple cell types can confuse analyses of host responses, especially if cultures include significant numbers of cells that differ in their susceptibility to infection or in their ability to support a persistent infection. Preliminary characterization of the differentiated cultures showed that few if any cells were positive for nestin, a progenitor marker, indicating that differentiation of the NSCs was efficient. Instead, the majority of cells expressed neuronal markers MAP2A and β-3 tubulin, and exhibited stereotypical neuronal morphology. Using a similar differentiation protocol and the parental H9 hESC line, Sadaoka and colleagues detected Brn3a/POU4F1, a marker of sensory neurons in 5% of the neurons in their cultures [42]. It will be interesting to determine the pattern of neurotrophin receptor expression, as this may also be an important determinant of the viral infection program.

In humans, latent HSV-1 can be readily detected in both sensory and autonomic ganglia at many different anatomical sites [72,73], suggesting that multiple neuron subtypes can support latency. The development of protocols for guided differentiation and direct conversion of human stem cells into defined neuronal subtypes is a very active area of research, but it remains difficult to obtain sufficiently homogenous cultures in quantity [74]. As these methods become robust and better understood, it will be interesting to directly compare different neuronal subtypes in terms of their ability to support persistent infections by different HSV-1 genotypes. Lastly, use of alternative reprogramming protocols that do not involve continuously dividing progenitors might reduce the time and effort required to produce usable neuron preparations [75].

Other laboratories are also exploring the use of human neurons generated in vitro as tools to study HSV-1 and VZV [42,76,77,78]. Whilst this manuscript was in preparation, Thellman and colleagues described an alternative HSV-1 latency model based on neurons derived from an immortalized human DRG cell line designated HD10.6 [79]. Proliferation of the cell line is driven by the expression of a v-myc oncogene. When this is prevented, the cells stop dividing and adopt a sensory neuron-associated phenotype, termed SNAP. Infection of SNAP cultures using strain KOS or 17syn^+^ reporter viruses in the presence of ACV, added 1 h after infection, was sufficient to establish a non-productive infection resembling latency within four days. However, reactivation in response to a variety of insults, including exposure to the deacetylase inhibitor trichostatin A was modest, barely exceeding that of untreated controls. Only a combination of NGF depletion and superinfection with UV-inactivated virus produced a measurable increase in reactivation relative to spontaneous reactivation. Whether the stability of the infections reflects the low levels of LAT RNA in this model, genotypic modifications that occurred during immortalization or passage of the HD10.6 line, redundancy in neurotrophin signaling or some other parameter needs to be determined. Regardless, the availability of several infection models, each conferring different biological properties on the virus, may prove to be an asset for the field by mimicking the diversity of neuron subtypes in the human nervous system and context-dependent behavior of the virus. Comparing and contrasting the different models will provide clues to the various host regulatory pathways that influence latency and reactivation and may explain some of the known, but puzzling, redundancy in viral factors.

## 4. Materials and Methods

### 4.1. Generation of Human Neural Stem Cells and Differentiated Neurons

Human embryonic stem cell (hESC) line Hes5::GFP [45], a derivative of the NIH approved H9 line, was maintained as colonies in hESC media (DMEM/F12 (Invitrogen, Carlsbad, CA, USA), 20% KnockOut Serum Replacement (Invitrogen), 5 ng/mL FGF2 (R and D Systems, Minneapolis, MN, USA), 2 mM glutamine (Invitrogen, Carlsbad, CA, USA), 0.1 mM non-essential amino acids (Invitrogen), 0.1 mM β-mercaptoethanol (Invitrogen) on a layer of mouse embryonic fibroblasts (MTI-GlobalStem, Gaithersburg, MD, USA) plated 24 h earlier on 0.1% (*w*/*v*) gelatin-coated plates [42]. Cultures were routinely groomed to discard differentiating colonies and the media was changed daily. Passaging was performed using dispase (Invitrogen) or by manually picking colonies. Differentiation into neural stem cell (NSC) rosettes was performed using the serum-free embryoid body method of differentiation [42,43] and addition of 100 ng/mL noggin (R and D Systems) and 10 μM SB431542 (Tocris Bioscience, Bristol, UK), a selective inhibitor of TGFβ receptor I. NSC generation from Rosette-NSCs was modified and adapted from [41,44]. Rosette-NSCs were picked by hand based on GFP fluorescence, dissociated in trypsin, and plated at 100 × 10^5^ cells/cm^2^ in neural stem cell media (NSCM) comprising N2 media (Invitrogen) supplemented with 20 μg/mL insulin (Sigma-Aldrich, St. Louis, MO, USA), 1 μL/mL B27 (Invitrogen), 1.6 g/L glucose (ThermoFisher Scientific, Waltham, MA, USA) and 20 ng/mL EGF and FGF2 (R and D Systems) on poly-l-ornithine and laminin-coated plates (Sigma-Aldrich). Cells were passaged at a ratio of 1:2 and maintained at high densities for 30 days before being used for experiments. For terminal differentiation into neurons, NSCs were plated at 2.5 × 10^5^ cells/cm^2^ on poly-l-ornithine/laminin-coated dishes and maintained in NSCM for 24 h, after which the culture media was changed to N2 media supplemented with 20 ng/mL brain-derived neurotrophic factor (BDNF, R and D Systems) and 0.2 μM ascorbic acid (Sigma-Aldrich). Media and growth factors were replaced every two days for 30 days before analysis or viral infection.

### 4.2. Indirect Immunofluorescence Analysis

Neuronal stem cells and differentiated neurons were fixed with 4% paraformaldehyde and probed using antibodies to nestin (MO15012, Neuromics Inc., Minneapolis, MN USA), microtubule-associated protein 2A (MAP2A, mab 3418, Millipore, Billerica, MA, USA), β-3 tubulin (Tuj1, MRB-435P, Biolegend, San Diego, CA, USA), and doublecortin (DCX, sc-8066, Santa Cruz Biotechnology, Dallas, TX, USA). Nuclear DNA was visualized by staining with 4′,6-diamidino-2-phenylindole (DAPI, ThermoFisher Scientific).

### 4.3. Infection of hESC-Derived Neurons with HSV-1

Neuron cultures were infected with either wild-type HSV-1 (strain Patton) or a recombinant type expressing enhanced GFP fused to the N-terminus of the viral true-late gene Us11 [52]. Viruses were prepared and titered on Vero cells by standard methods. To establish non-productive infections, combinations of the viral replication inhibitor acyclovir (Sigma-Aldrich) at 100 μM and human IFN-α (50 to 500 IU/mL, Novus Biologicals, Littleton, CO, USA) were added to the neuron cultures one day before infection. For infection studies, neurons (10^5^/well) were maintained in 24-well culture dishes and infected by adding diluted virus directly into the maintenance media. After 4 h, the maintenance media was replaced with fresh media and changed every other day. ACV and IFN were included in the media for up to six days post-infection. Likewise, in the absence of antivirals the media was changed every two days and the cultures monitored daily for GFP-Us11 accumulation by fluorescence microscopy. Each assay condition was scored across at least 12 separate wells. 

### 4.4. RNA Isolation and Transcript Analysis by Quantitative Reverse Transcription PCR (qRT-PCR)

Total RNA was extracted using Trizol (Life Technologies, Carlsbad, CA, USA) and precipitated with ethanol. Following treatment with DNase I (Promega, Madison, WI, USA), phenol-CHCl_3_ extraction, and ethanol precipitation, RNA yield and concentration were determined by absorption spectroscopy using a NanoDrop (NanoDrop Technologies, Wilmington, DE, USA). All samples were prepared in three biological replicates. RNA (300 ng/sample) was used to generate cDNA with Superscript III (Invitrogen) and random hexamer primers (Fermentas, Waltham, MA, USA). Levels of selected viral lytic mRNAs were quantified by qRT-PCR using the following primer sets:

GAPDH FW: 5′-TCTTTTGCGTCGCCAGCCGA-3′
GAPDH RV: 5′-ACCAGGCGCCCAATACGACC-3′
LAT FW: 5′-AGTCCGGGCGGGCAGGCGCT-3′
LAT RV: 5′-GCCCGGGCTGCCTGACCACCGAT-3′

Real-time qPCR analysis was performed using FastStart Universal SYBR Green Master-ROX (Roche, Indianapolis, IN, USA) and a MyiQTM single-color real-time thermal cycler (BioRad, Hercules, CA, USA). Relative changes in transcript levels were calculated using the ΔΔCt method.

### 4.5. Reactivation and Determination of Viral Titer

To induce reactivation, 5 mM sodium butyrate (Sigma-Aldrich) was added the culture media and maintained until green fluorescence indicative of productive replication was detected. Viral titers were determined by plaque assay on Vero cell monolayers using serial dilutions of culture media collected from individual wells.
